# Creosote for Bronchitis and Catarrh

**Published:** 1881-01

**Authors:** 


### CREOSOTE FOR BRONCHITIS AND CATARRH.

When going from Switzerland to Italy, via Mount Cenis, some years ago, the writer contracted a sudden severe cold, which, in the chill air of Turin, soon brought on a severe attack of bronchitis. We hastened over to the genial air of Genoa, but it afforded little relief, and the advice of Dr. Paccioci, professor in the noted Italian medical college there, was called in. He prescribed a very simple remedy, which was at once effective, as it has been with many others to whom we have since reccommended it. Put into a pint or larger bottle about three gills of water, and add two drops of good creosote. Shake very thoroughly, take a mouth-full, gargling it awhile in the throat, and swallow it. Repeat this frequently, so as to use up the mixture in the first twenty-four hours, always shaking well before taking. After the first day use three drops of creosote, and the same amount of water during twenty-four hours, so long as it is needed.

The same mixture has often proved very useful in catarrh. In this case a handful or two of the well-shaken creosote and water is snuffed up through the nostrils until it reaches the throat and is spit out. A tablespoonful is also gargled in the throat and swallowed. As catarrh is an inflammation of the nasal passages, accompanied with a mucus deposit, the creosote, which is largely carbolic acid, would seem to be useful here, just as dilute carbolic acid is effective in cleansing any putrid sores. Catarrh is the result of weakness, and is promoted by a cold. A toning up of the system and any simple remedy like the above is effective, unless the catarrh is severe and of so long continuance as to have permanently disorganized the nasal cavities. It is folly to spend money for the much advertised catarrh remedies, which are usually the sheerest medical quackery.—*American Agriculturist.*

### FEMALE DRESS IN ANCIENT TIMES.

In the wardrobe of a Hebrew lady the most splendid article of clothing was the turban, for those who could afford it. The poor people had to be satisfied with winding a piece of cloth round their head, and fixing it as well as they could. The turbans were of various colors and wound in different ways ; some of them were like a high tower. Shoes and stockings were unknown, but soles of leather were fastened with two latchets. The ladies who carried luxury into every department, and who are supposed, even in the present day, to be far from indifferent to a nice, neat boot, or to elegant slippers, had their shoes, or rather sandals, and their latchets, made of colored leather ; dark blue, violet and purple were favorite colors. The ankles were decorated with bracelets of gold or dainty silver chains and rings, with tiny silver bells. Hairnets and headbands were in great request. The latter were of gold and silver, and worn under the net, extending from one ear to the other. Ear-rings were much thought of ; we are told of some that weighed a thousand and seven hundred shekels of gold, and were so large that a man could easily put his hand through them. Some of the women wore several rings with little bells attached to them. They were generally made of horn or of silver. But the most popular ring was the nose-ring. The left nostril was pierced for the purpose, and a ring made of ivory or metal put through it. Bracelets were favorite ornaments, and generally worn on the right arm. Some of them were exceedingly large, so that they reached up to the elbow. Rings on the fingers were not worn ; chains of fine gold, or strings of pearl, with little silver balls, or small tinkling bells, were worn round the neck.

**KNIGHT BROTHERS,**

SOLICITORS OF

UNITED STATES AND FOREIGN

**PATENTS,**

**Counselors in Patent Causes**

AND

**MECHANICAL EXPERTS,**

**AGENCIES AT PRINCIPAL FOREIGN CAPITALS.**

**No. 633 F Street, N. W., Washington, D. C.;**

**75 West Fourth Street, Cincinnati, Ohio;**

**317 Olive Street, St. Louis, Mo.**

**SEND FOR BOOK OF INSTRUCTIONS.**

### DR. HALL'S PORTABLE SPIROMETER.

NO PHYSICIAN questions the value of the spirometer for exercising and expanding the lungs, and for measuring their capacity. The great difficulty has been to find an instrument that can be compressed into a small compass when not in use, so that the physician or patient may conveniently carry it from place to place as circumstances require. Another obstacle to the more general use of this truly valuable instrument has been its price. The unwieldy spirometer of a few years ago cost from $10 to $20. Dr. Hall's Portable Spirometer sells for $7.50, and will be sent free to any address on receipt of the price by the editor of this Journal.

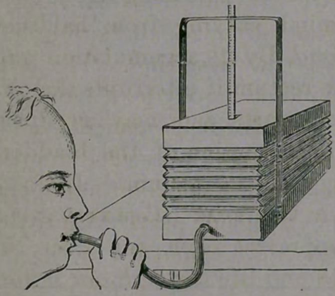

It is 6 by 12 inches, and when closed it stands only 3 inches high, and weighs less than 3 pounds.

This cut represents Dr. Hall's Portable Spirometer, partly inflated, in order to show the manner of using it. When not in use it can be closed together so as to occupy the small space above stated. The scale and guide are hinged, so that they close down on the top of the Spirometer, and occupy but little space. The top is of polished black walnut. The brass-work is plated with silver or nickel.

HOW TO USE THE SPIROMETER.

The instrument should be used only in a perfectly ventilated room which is entirely free from dust, or else in the open air, away from the dust and smoke and bad odors of the streets of a city. When it is remem, bered that the capacity of the lungs of a man of medium size is from 150 to 250 cubic inches, and that in the act of breathing naturally, or rather as some have acquired the habit of breathing—which is far from natural —the lungs take in only from 20 to 40 cubic inches of air at each inspiration, leaving from 130 to 230 cubic inches of air cells almost always in a dormant condition, the importance of regular and systematic exercise for the lungs will be appreciated. But, like all good things, this sort of lung exercise must be indulged in moderation. Draw a full breath and then blow through the rubber tube into the spirometer; at the same time watch the figures on the scale as it rises. The figures seen on the scale as it passes through the top of the guide indicate the number of cubic inches of air blown into the spirometer.

It is now very generally conceded that mankind start quite evenly on the journey of life, in spite of supposed hereditary tendencies to pulmonary consumption, and that with proper care the average term of life is not shortened by these hereditary tendencies. This being the case, it is far better to enjoy health and long life through means which the naturally robust are not compelled to resort to, than to believe in or lament over the inheritance of ills that do not exist. We believe for all persons who are confined much of the time in doors, particularly in cases where there is a tendency to weak lungs, the regular use of the spirometer in the open air is highly beneficial. Its use just before bedtime is said to induce sleep, by drawing blood from the brain to the lungs.

### CONSTIPATION.

CONSTIPATION becomes alarmingly frequent as people acquire the means of living luxuriously without regular manual labor. It is still more frequent among persons who become so through ignorance of the penalties that follow a want of attention to the regular demands of nature.

When fecal matter is retained in the rectum more than twenty-four hours, it commences its destructive work, notably in two ways. *First*, It is absorbed into the system, and acts as a blood poison. One of the worst results of blood poisoning from constipation is to cause disease of the mind, varying from habitual moroseness to actual insanity. *Second*, By its accumulation and impacting in the lower portion of the rectum it interrupts the circulation of the blood in the adjoining parts, and may produce, from this cause alone, piles, fistula, inflammation of the bladder, disease of the prostate gland, or cancer of the rectum. In fact, most of these diseases are developed in this way. To a very great extent we hold our health and happiness, even our lives, in our own hands. If we sacrifice health and life through our ignorance, or indifference, we pay the penalty through suffering, more severe because more prolonged than instant destruction. From whatever cause, there is, unfortunately, great ignorance in regard to the laws of health among a class of people who are otherwise intelligent. How many parents are there who look carefully to the habits of their children, upon which all that will make life desirable for them so much depends? Cathartics and clysters should not be used in constipation. They increase the trouble.

That which has, perhaps, met with most general favor relates to the diet. Certain foods have been regarded as specifics for this complaint, and particularly "Graham bread." This often induces activity of the bowels, but it ruins digestion like other cathartics, in a manner which we have heretofore fully explained in the pages of this JOURNAL. Kneading the bowels has sometimes afforded relief, but it affords no permanent benefit, as a rule. We have, however, found a remedy for constipation and for piles which is always safe, and is attended with no inconvenience or discomfort. We have prescribed it in hundreds of cases. It has usually afforded prompt relief, and many patients who had suffered for years believe that they have been permanently cured of constipation by its use. This remedy is "Nelaton's Suppository." It is prepared and sold by Hall & Ruckel, wholesale druggists, 218 Greenwich street, New York, at 50 cents and $1.00 a box, and is sent free by mail on receipt of price.

